# Knowledge and Attitudes towards Epidural Analgesia among Women of Childbearing Age in Jazan, Saudi Arabia: A Community-Based Cross-Sectional Study to Identify Predictors through Multivariate Modeling

**DOI:** 10.3390/healthcare11040626

**Published:** 2023-02-20

**Authors:** Yasir Osman Hassan Babiker, Muhannad Hussain Shawkan Najmi, Ibrahim Mohammed A. Muslihi, Ali Fathuldeen Mohammed Amri, Tariq Yahya Mohammed Magafi, Wail Mohammad Hadi Alughbi, Mohammad Refah A Bashir, Ali Nasser Ahmed Alsharif, Anas Elyas Ahmedand, Siddig Ibrahim Abdelwahab

**Affiliations:** 1 Department of Surgery, College of Medicine, Jazan University, Jazan 45142, Saudi Arabia; 2 Department of Family and Community Medicine, College of Medicine, Jazan University, Jazan 45142, Saudi Arabia; 3 Medical Research Centre, Jazan University, Jazan 45142, Saudi Arabia

**Keywords:** epidural analgesia, labor pain, childbearing age, Saudi Arabia, modelling

## Abstract

Epidural analgesia (EA) is a central nerve blockade technique. It is linked to a significant reduction of labor pain and side effects. This study was designed to investigate the knowledge and attitudes towards EA among women of childbearing age (18–45 years) in Jazan, Saudi Arabia, and identify predictors through multivariate modeling. A random sampling technique (n = 680) was used for this cross-sectional, self-administered survey. A previously validated online questionnaire was distributed. After establishing a P value of less than 0.05 to denote statistical significance, SPSS was used to examine the data using descriptive analysis, the chi-square test of homogeneity, and multivariate logistic regression. Six hundred and eighty women were studied. Over 75% of the participants were university educated; less than half (46.3%) were 21–30 years old, students (42.2%), and had never been pregnant (49%). The previous mothers who had never had EA labor accounted for 64.6% (n = 347, 51.0%). “Family/friends” (39%), followed by “internet” (32%), were the most common sources of EA information. Those who correctly defined the EA accounted for 61.8%. Those who reported weak or no contractions after EA accounted for 32.2%. Those who said EA insertion hurt more than labor did accounted for 56.3%. Those women who said one should give consent to EA accounted for 83.1%. Those who believe EA is safe for the baby accounted for 50.1%. Those who knew about EA complications accounted for 24.34%. According to multivariate modeling, attitude score plays a significant role in determining the participant’s knowledge level. This study found that childbearing women know a little about EA. Attitudes affected this knowledge level, and demographics did not. Cognitive intervention is needed to change these attitudes and spread EA-related knowledge.

## 1. Introduction

Epidural analgesia (EA) were introduced to obstetrics in 1946. In recent years, there has been a worldwide rise in the number of birthing women who turn to EA for pain management during childbirth. Epidural labor pain medication has become increasingly popular among women in the United Kingdom, the United States, and China over the past 20 years. The majority of nations practice EA. There is a significant gap in its application from one healthcare system to the next, and even within countries, there is diversity. It is estimated that anywhere from 50% to 90% of all obstetric units make use of EA as their primary method of providing labor analgesia. This practice is more common in nations with higher per capita income. This stands in stark contrast to the results that are obtained from nations with limited incomes, where between 1.3 and 12% of parturients receive pain relief via epidural analgesia [1,2,3,4].

This type of anesthesia works by shutting down the central nervous system. An anesthetic is injected into the epidural area of the lower spine, which blocks the nerves that transmit pain from the uterus and birth canal. This approach provides intraoperative and postoperative pain management. The spinal EA quickly reduces the effect of the local anesthetic and improves dural puncture and sacral spread [5,6,7]. Anesthesiologists turn to EA as their first line of defense against the discomfort associated with postoperative recovery and as the major analgesic. This topical technology not only provides good pain relief, but it also reduces the exposure time to anesthetics and other analgesics, which in turn reduces the risk of side effects. It decreases the cortisol levels, improves bowel function, lowers the risk of pulmonary embolism and deep vein thrombosis following surgery, and shortens the amount of time spent in the hospital [8,9]. Treating labor pains through EA can impair breastfeeding and mother–child communication [10]. EA may also affect the mother and her baby during childbirth. EA can also cause hypotension, fever, and a prolonged labor. It may also cause dystocia [11,12]. EA increases the need for oxytocin, prolongs the second stage of delivery, and causes the malposition of the posterior occipital in the fetus [13,14,15]. 

EA has improved instrumental delivery in various studies [16,17,18]. Other studies have linked EA to cesarean delivery [19,20]. After neuraxial analgesia, 10-20% of patients’ fetal heart tones were unsettling, although this did not harm the infant [21]. The difference in hypertonic uterine contractions following spinal opioid and epidural injections may be explained by analgesia’s rapid fall in plasma epinephrine [5]. EA does not lengthen the labor when it is compared to the duration of labor without it. To maintain the typical length of labor, however, substantial oxytocin supplementation is required during EA [3,14].

Due to the method’s introduction in the latter half of the 20th Century, its subsequent spread as a clinical practice among obstetrics departments, and the benefits and drawbacks that are connected to it, it became necessary to learn about women’s knowledge of the technique, as well as their behavior regarding it. These elements necessitated learning about women’s proficiency with the procedure. Women’s perceptions on and awareness of EA have been the subject of prior study conducted across the globe [22,23,24]. Since a recent study was conducted on women who had already given birth, there have been no studies conducted locally on women of reproductive age in the Jazan region [25]. However, this study, such as others, focused on all age groups of women of reproductive age. Community outreach initiatives that are successful must start soon. Accordingly, the current study aims to measure the knowledge and behavior of Saudi women of childbearing age in the Jazan region regarding EA. It also analyzed of factors affecting knowledge through using logistic regression.

## 2. Materials and Methods

### 2.1. Study Design, Area, and Population 

The province of Jazan, which is located in Saudi Arabia, served as the research location for this cross-sectional study. Adult females ranging in age from 18 to 45 years were sought for participation in the study. The area known as Jazan can be found in the southwestern corner of Saudi Arabia, directly to the north of the international border with Yemen. According to some estimates, there are approximately 1,600,000 people living there.

### 2.2. Inclusion and Exclusion Criteria

Adult Saudi Arabian females between 18 and 45 who were residents of the Jazan region participated in this study. Additionally, every single woman who could read and had a sincere interest in participating in this research was included. Participants who suffered from mental impairments were not allowed to take part. Women under the age of 18, women who were over the age of 45, and women who did not live in Jazan, Saudi Arabia, were excluded from this study.

### 2.3. Sample Size and Sampling Technique 

The size of the sample was determined with the help of the EPI info tool. Whereby, the following formula was applied:

n = [1.96^2^ ∗ P(1 − P)]/α^2^

It was calculated using a confidence interval of 95%, a P of 0.5, a margin of error of 5%, and the total population sampled in Jazan, Saudi Arabia. The projected size of the sample was 680. However, this number was increased to 748 to account for a 10% non-response rate among the total population sampled in Jazan, Saudi Arabia. A random sampling technique was employed to collect the data from the participants.

### 2.4. Data Collection Tools

The study was conducted using an online, self-administered questionnaire via Google Forms. The generated link was randomly shared using online platforms. A validated questionnaire was used based on previous studies [23,24,26]. The questionnaire contained socio-demographic characteristics of the participants, such as age group, sex, nationality, and residence. The questionnaire also included two scales for knowledge and attitude toward EA. The reliability of both scales was checked using Cronbach’s alpha, which was found to be greater than 0.7. The attitude score was established using the summation formula. This study utilized knowledge as a dependent variable to identify the factors that influence women’s knowledge. The final score was based on the previously reported method [27]. Each respondent’s correct responses were tallied and gathered to obtain a result, with the lowest score being zero and highest score being twelve. The respondents were divided into two groups: those with limited knowledge, who received scores below 6, and those with good knowledge, who received scores above 6. The data were collected between December 2021 and July 2022.

### 2.5. Data Analysis

The 23rd edition of the Statistical Package for the Social Sciences (SPSS) was utilized for the data coding, entry, and analysis. The primary outcome is knowledge prevalence. The results of the qualitative data were presented as numbers of occurrences and percentages (N and %, respectively). The chi-square homogeneity test was used to determine whether frequency counts are distributed identically across different populations. Multivariate logistic regression modeling was used to understand the relationship between knowledge and other variables as dependent and independent variables. The independent variables included in the model were age, education level, occupation, pregnancy, past labor with EA, source of information, and attitude score. Levels of significance, adjusted odds ratios (OR), and confidence intervals (CI) were used to determine the association between the dependent and independent variables. The Hosmer–Lemeshow test confirmed that the data used in the current study fit the logistic regression model.

### 2.6. Ethical Considerations

The Research Ethics Committee of Jazan University provided their consent for the project, which was then formally approved (HAPO-10-Z-001). All of the data were kept in strict confidence and were solely applied to the study being conducted.

## 3. Results

A total of 680 women participated in the study, corresponding to a 100% response rate. Over 75% of the participants were university educated or above, while less than half of the participants (46.3%) were within the age group 21–30 years, students (42.2%), and had never been pregnant before (49.0%). Among those who had given birth before (n = 347, 51.0%), about 64.6% had never experienced a past labor with EA. More details are presented in Table 1. 

**Figure 1:**
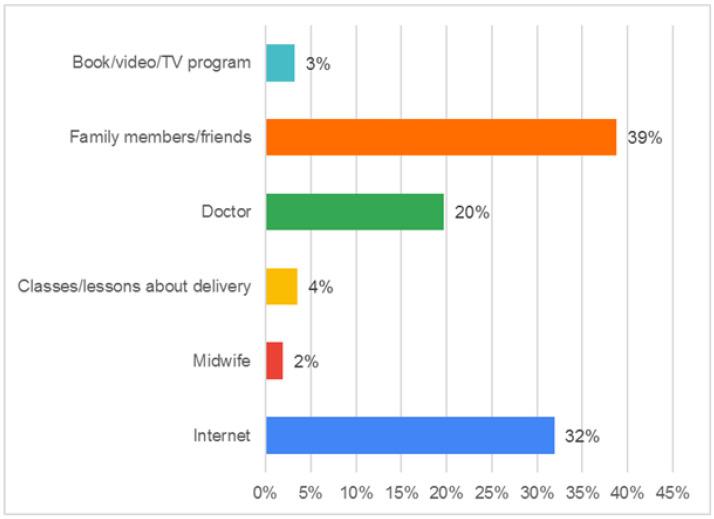
Source of information about EA as reported by the study participants.

The most frequent source of knowledge about EA, according to participants, was “family/friends” (39%), followed by “the internet” (32%), and “the midwife”, which was the least preferred option (2%). There is more information in Figure 1. To check if the frequency counts were distributed uniformly across population subgroups, the chi-square homogeneity test was utilized. We rejected the null hypothesis because the *p*-value (*p* = 0.00006) was lower than the significance level (0.05).

Table 2 shows the knowledge responses of the participants. Only 61.8% of the participants answered “yes” for the definition of the EA (Q1), but 74.6% answered “no” for Q2, if any physician or nurse may administer it. Some women (32.2%) chose “yes” for contractions to weaken or cease after EA (Q3), while 46.9% were undecided. More than 65% of the participants answered “yes” to Q4, “EA is the most frequently utilized and most effective technique of alleviating labor pain”, whereas 52.5% were unsure if EA increases the C-section risk (Q5). More than half of the women (56.3%) said EA insertion is more painful than labor discomfort is (Q6), while 31.5% were undecided. Q7: Does EA minimize labor pain and allow the woman to push? More than half of the women (59.3%) said “yes”, and 31.9% were undecided. Most of them (83.1%) said women should consent to EA during childbirth (Q8). More than half of them (50.1%) though that EA is safe for the baby (Q9), while 43.1% were unclear about this. Surprisingly, 12.9% of the participants answered “no” to Q10 about EA complications (headache, fever, and lower blood pressure for the mother), while 59.0% were undecided. Those respondents who did not think EA might induce muscle weakness in the mother’s lower limbs accounted for 11.6% (Q11), while 59.7% were unclear about this. EA should be a choice for women during birth (Q12) according to 68.1% of respondents, while 10.6% disagreed. The average knowledge score is 7.00 ± 2.46.

Table 3 indicates the participants’ attitude answers. The majority of the individuals (84.9%) thought that childbirth is uncomfortable (Q1), 75.4% agreed that EA relieves childbirth pain (Q2), and 46.0% said that they will have an EA during their current pregnancy or the next one (Q3). Most (84.6%) of the participants preferred if a doctor introduced and explained EA during pregnancy at follow-up clinics (Q4), whereas 39.4% think the doctor’s gender will influence EA decision making (Q5). Only 41.9% disagreed that the community needs more EA awareness and advice (Q6). The average attitude score is 21.84 ± 3.48. A chi-squared test of association showed a significant association between age and items 3, 5, and 6 (Table 3). 

To understand the relationship between knowledge and the dependent variables and independent variables, multivariate logistic regression modeling was used. Age, education level, occupation, pregnancy, prior EA work, information source, and attitude score were the independent variables in the model. The link between the dependent and independent variables was ascertained using the level of significance, an adjusted odds ratio (OR), and its confidence intervals (CI). The raw and adjusted odds ratios (Table 4) for all of the independent factors fluctuated between increases and decreases, indicating that the multifactorial modeling affected the knowledge of EA. In contrast to the other independent variables, it was discovered that women’s degree of knowledge was statistically (*p* < 0.05) connected to their attitude score. This was determined based on the significance level. In this particular investigation, the level of knowledge was not significantly affected by any of the other independent factors that were considered (Table 4).

## 4. Discussion

It is crucial to evaluate the level of knowledge and the attitudes regarding EA since it impacts maternal decisions and, as a result, the well-being of the mother and her child (33). This research aimed to investigate women’s perspectives concerning the use of EA in the Jazan Region of Saudi Arabia. No previous studies in the Jazan region included women of childbearing age. Only one study in the Jazan region was conducted on pregnant women [25]. However, a study on Saudi women of childbearing age’s knowledge of EA during standard vaginal delivery was carried out in the Khamis Mushait region [26]. This study was not a community study such as ours is; instead, it was conducted in a single primary health center with women who gave birth naturally. No matter the type of birth, all women were eligible for the current study. 

More than two-thirds (75%) of the participants were university educated or above. Less than half of the participants (46.3%) were 21–30 years old. Additionally, less than half (42.2%) of them were students. Almost half of them (49.5%) have never been pregnant before. Among those who gave birth before (51.0%), about two-thirds (64.6%) of the participants never had a previous labor with an EA. The most reported source of information about EA was found to be “family members/friends”, as stated by 39% of the participants, followed by “the Internet”, as mentioned by 32%, and this was found to be in contradiction to the study conducted by Stamer et al., in which information leaflets and posters were the most reported source of information among the study participants [28]. A recent study in the Jazan region showed that the source of women’s knowledge was the healthcare staff [25]. This disparity in the level of awareness and acceptance of EA in labor could be explained by the fact that in developing countries, childbirth is viewed as a physiological process that does not require much interference. As a result, the levels of awareness and acceptance of EA in the labor are lower in these countries. The low degree of knowledge is also attributable to the absence of a prenatal follow-up and explanations during antenatal sessions.

In a Pakistani study by Minhal et al., 76% of the participants knew the definition of EA [23], rather than 61.8% in our study. A Nigerian study found that 40% of women had knowledge of EA [29]. Those physicians and nurses who reported EA administration difficulties accounted for 74.6%. After EA, 32.2% of individuals reported weak or no contractions. EA decreases or stops uterine contractions in 26% of the subjects, which is the same as that in Ageel et al.’s study [25]. EA is the most common and effective labor pain reliever, according to 65% of the participants. In the parallel research by Naithan et al., only 4% of the participants claimed that EA increases the chance of a C-section, but 16% of the people in this study claimed this [24]. In contrast to Gari et al., more than half (56.3%) of the participants felt that EA insertion was more painful than labor discomfort was [30]. Those participants who claimed that EA decreases labor discomfort and lets the mother push account for 59.3%. Those who said women should consent to an EA during labor account for 83.1%. In a comparable study by Alahmari et al., most of the participants thought that EA reduced labor discomfort and agreed that informed consent was crucial [26]. About half (50.1%) of the participants do not feel EA is risky for the baby. Almost 24.3% of the participants knew about the complications (EA can cause headaches, fever, and lower blood pressure in the mother); this percentage is similar to that which was reported in a similar study conducted by Mccauly et al., in which 20% of the participants were informed about the complications of EA [22].

Multivariate logistic regression modeling was used to understand knowledge through dependent and independent variables. Age, education, occupation, pregnancy, prior EA work, information source, and attitude score were independent variables in the model. The raw and adjusted odds ratios for all of the independent factors varied, suggesting that multifactorial modeling influences EA knowledge. Unlike the other independent factors, women’s knowledge was significantly (*p* < 0.05) linked to their attitude score. None of the independent factors in this study affected knowledge (Table 4). Age was not found to be significantly associated with knowledge. The present findings contradict the findings of the research conducted by Minhas [23]. However, they were in line with the results published by Gari et al. [30].

Two earlier studies, one conducted in India [24] and the other one conducted in Saudi Arabia [31], revealed that a good understanding of EA is related to the level of EA experience women have had in the past. In contrast, these results were inconsistent with the current study’s findings. This gap can be explained by the fact that both sets of analyses were conducted on pregnant or previously pregnant women, rather than our research, which was conducted on women of childbearing age.

There are certain restrictions on the current investigation. First, it is impossible to determine the response rate because we have less control over who receives a copy of the survey using this method of data collecting. Second, it is only available to Internet users. Third, the discussed connections and risk factors should be carefully taken into account because this is a cross-sectional study. Fourth, only female individuals who could read were examined in this study. Despite the fact that this is a sizable demographic, it did not include mothers who could not read. In a cross-sectional research design, the exposure and the result are both assessed at the same time, hence there is often no indication of a temporal relationship between the two variables. The fifth and last drawback of the cross-sectional study approach is this. If longitudinal data are not analyzed, it is impossible to establish a true cause-and-effect link.

## 5. Conclusions

The participants had an average level of understanding of both the process and the goal of EA. The majority of respondents knew the importance of obtaining consent before conducting an EA. Concerning the complexities of EA, there are gaps in people’s awareness. It is advised that a health education program be established to convey information about EA to all females in the reproductive phase and to meet the needs of women who are interested in learning more about EA. These awareness programs must incorporate observation, communication, and a straightforward schematic of the EA method to make it easier for obstetricians and anesthesiologists to collaborate. These findings can be relied upon by medical professionals worldwide when they are building knowledge programs to improve reliance on them by raising health awareness and using this anesthetic method. In contemporary obstetrics, efforts must be made to raise awareness, debunk common myths, and subsidize the costs of delivering this valuable quality of care. One of the main findings of this study is that attitudes are a significant predictor of the level of knowledge. Therefore, future studies are recommended to identify trends through qualitative studies.

## Figures and Tables

**Table 1:** Socio-demographic characteristics of the study participants.

	Frequency	Percentage
**Age group**		
<20 years	95	14.0
21–30 years	315	46.3
>30 years	270	39.7
**Education level**		
Primary/Intermediate	15	2.2
Secondary/Diploma	149	21.9
University and above	516	75.9
**Occupation**		
Unemployed	210	30.9
Student	287	42.2
Employed	183	26.9
**Parity pregnancy**		
Never	333	49.0
Primigravida	75	11.0
Multigravida	272	40.0
**Past labor with EA (N = 347)**		
No	224	64.6
Yes	123	35.4

**Table 2:** Response to knowledge questions for the whole samples.

Statement	No	Not Sure	Yes
	N (%)		
Q1. Epidural Analgesia is an injection of local analgesia through a catheter into the epidural space of the spine.	25 (3.7)	235 (34.6)	420 (61.8)
Q2. Any physician or nurse can administer the Epidural Analgesia.	507 (74.6)	122 (17.9)	51 (7.5)
Q3. Contractions become weak or stop completely after administration of Epidural Analgesia.	142 (20.9)	319 (46.9)	219 (32.2)
Q4. Epidural Analgesia is the most frequently used and most effective way of relieving labor pain.	54 (7.9)	178 (26.2)	448 (65.9)
Q5. Epidural Analgesia increases the risk of having a C section.	214 (31.5)	357 (52.5)	109 (16.0)
Q6. The Epidural Analgesia insertion is more painful than labor pain itself.	383 (56.3)	214 (31.5)	83 (12.2)
Q7. Epidural Analgesia reduces labor pain and allows the mother to push when needed.	60 (8.8)	217 (31.9)	403 (59.3)
Q8. Women should agree and provide consent for having Epidural Analgesia at labor.	15 (2.2)	100 (14.7)	565 (83.1)
Q9. Epidural Analgesia is risky for the baby.	341 (50.1)	293 (43.1)	46 (6.8)
Q10. Epidural Analgesia can cause headache, fever, and lower blood pressure of the mother.	88 (12.9)	401 (59.0)	191 (28.1)
Q11. Epidural Analgesia can cause muscle weakness in the lower limbs of the mother.	79 (11.6)	406 (59.7)	195 (28.7)
Q12. Epidural Analgesia should be an available option for women at delivery.	72 (10.6)	145 (21.3)	463 (68.1)
Knowledge score	7.00 ± 2.46		

**Table 3:** Response to attitude questions for the whole samples and their association to age.

Items	Strongly Disagree	Disagree	Neutral	Agree	Strongly Agree	*p*-Value *
Q1. If you have tried giving birth at least once, do you think childbirth is painful?	6.0 (0.8)	31.0 (4.4)	79.0 (11.1)	249.0 (35.0)	346.0 (48.7)	**0.380**
Q2. Do you think using EA help relieve childbirth pain?	13 (1.8)	51 (7.2)	119 (16.7)	337 (47.4)	191 (26.9)	**0.056**
Q3. I will order EA during the birth of this pregnancy or the next pregnancy.	46 (6.5)	128 (18.0)	204 (28.7)	190 (26.7)	143 (20.1)	**0.00**
Q4. If you are seriously considering EA, would you prefer to introduce it and explain it in pregnancy follow-up clinics?	46 (6.5)	128 (18.0)	204 (28.7)	190 (26.7)	143 (20.1)	**0.065**
Q5. The gender of the doctor (male or female) has an influence on you in one way or another in decision making?	16 (2.3)	23 (3.2)	76 (10.7)	237 (33.3)	359 (50.5)	**0.00**
Q6. Adequate awareness and guidance about the use of pain relievers by using EA in our community is sufficient?	86 (12.1)	155 (21.8)	186 (26.2)	167 (23.5)	117 (16.5)	**0.00**
The attitude score	21.84 ± 3.48					

* Chi-squared test of association. Level of significance was set as 0.05. * Values less than 0.05 is considered significant.

**Table 4:** Univariate and multivariate logistic regression.

Variables	Crude OR	Adjusted OR	95% C.I. For OR	
			Lower	Upper
Age				
<20 Yrs	0.66	0.99	0.39	2.50
21–30 Yrs	0.86	1.31	0.67	2.57
>30 Yrs (REF)				
Education Level				
Secondary	0.35	0.43	0.08	2.26
University	0.34	0.39	0.08	1.93
Postgraduate (REF)				
Occupation				
Student	0.66	0.90	0.46	1.75
Employee	1.01	1.00	0.54	1.85
Unemployed (REF)				
Pregnancy				
Never	0.57	0.59	0.31	1.14
Primigravida	1.05	0.85	0.39	1.86
Multigravida (REF)				
Past labor with EA				
YES	2.42	1.69	0.85	3.37
No (REF)				
Source of Information				
Internet	2.38	2.43	0.51	11.46
Doctor	4.05	2.70	0.54	13.43
Family Member or Friends	2.80	3.25	0.69	15.32
Book or Videos	6.00	6.47	0.90	46.71
Midwife or Labor Classes	2.38	2.60	0.46	14.73
Society (REF)				
Attitude Score	0.00	2.20 *	1.60	3.03

*: reached level of significance: OR: Odds ratio; C.I.: confidence intervals; REF: reference group.

## Data Availability

Due to ethical consideration, authors are not able to share the data of the current research.
